# Benefits and Limitations of DNA Barcoding and Metabarcoding in Herbal Product Authentication

**DOI:** 10.1002/pca.2732

**Published:** 2017-09-14

**Authors:** Ancuta Cristina Raclariu, Michael Heinrich, Mihael Cristin Ichim, Hugo de Boer

**Affiliations:** ^1^ Natural History Museum University of Oslo P.O. Box 1172, Blindern 0318 Oslo Norway; ^2^ Stejarul Research Centre for Biological Sciences National Institute of Research and Development for Biological Sciences Alexandru cel Bun Street, 6 610004 Piatra Neamt Romania; ^3^ Research Group of Pharmacognosy and Phytotherapy, Research Cluster ‘Biodiversity and Medicines’, UCL School of Pharmacy University of London 29–39 Brunswick Sq London WC1N 1AX UK

**Keywords:** authentication, DNA barcoding, DNA metabarcoding, herbal pharmacovigilance, herbal products, NMR metabolomics, quality, safety

## Abstract

**Introduction:**

Herbal medicines play an important role globally in the health care sector and in industrialised countries they are often considered as an alternative to mono‐substance medicines. Current quality and authentication assessment methods rely mainly on morphology and analytical phytochemistry‐based methods detailed in pharmacopoeias. Herbal products however are often highly processed with numerous ingredients, and even if these analytical methods are accurate for quality control of specific lead or marker compounds, they are of limited suitability for the authentication of biological ingredients.

**Objective:**

To review the benefits and limitations of DNA barcoding and metabarcoding in complementing current herbal product authentication.

**Method:**

Recent literature relating to DNA based authentication of medicinal plants, herbal medicines and products are summarised to provide a basic understanding of how DNA barcoding and metabarcoding can be applied to this field.

**Results:**

Different methods of quality control and authentication have varying resolution and usefulness along the value chain of these products. DNA barcoding can be used for authenticating products based on single herbal ingredients and DNA metabarcoding for assessment of species diversity in processed products, and both methods should be used in combination with appropriate hyphenated chemical methods for quality control.

**Conclusions:**

DNA barcoding and metabarcoding have potential in the context of quality control of both well and poorly regulated supply systems. Standardisation of protocols for DNA barcoding and DNA sequence‐based identification are necessary before DNA‐based biological methods can be implemented as routine analytical approaches and approved by the competent authorities for use in regulated procedures. © 2017 The Authors. Phytochemical Analysis Published by John Wiley & Sons Ltd.

## Abstract 2

Herbal medicines play an important role globally in the health care sector and in industrialised countries they are often considered as an alternative to mono‐substance medicines. Different methods have varying resolution and usefulness along the value chain of these products. DNA barcoding can be used for authenticating products based on single herbal ingredients and DNA metabarcoding for assessment of species diversity in processed products, and both methods should be used in combination with appropriate hyphenated chemical methods for quality control.

## Introduction

**Figure 1:**
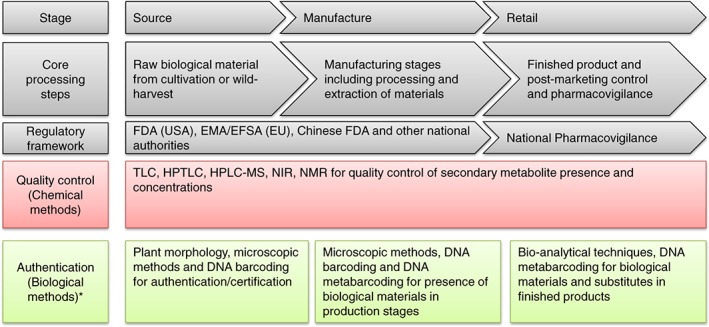
The different stages in the production of regulated or unregulated herbal medicinal products and the role of different analytical (chemical and biological) methods. *Methods used in quality control are also commonly used for authentication. [Colour figure can be viewed at wileyonlinelibrary.com]

Herbal medicines play an important role in many industrialised countries as a complement and alternative to synthetic pharmaceuticals. The global market for herbal products is projected to reach US$115 billion by 2020, with Europe leading the market (Global Industry Analyst, Inc., 2015). Their popularity is determined by consumers' health concerns, cultural factors, and the belief that these are natural and thus safe (Lynch and Berry, 2007; Ipsos MORI, 2008). Medicinal plants are sources of molecules with tremendous therapeutic potential and remarkable pools for novel drugs leads, but evaluating their safety, pharmacological effects and efficacy requires a thorough multidisciplinary scientific approach (Atanasov *et al*., 2015). An increasing awareness of quality irregularities is calling attention to the quality of traded mass‐produced herbal products with direct impact on their efficacy and safety (Heinrich, 2010; Leonti and Casu, 2013). Herbal product quality regulations vary between countries and together with a lack of standardised analytical methods (Locatelli *et al*., 2014; Locatelli and Celia, 2017; Melucci *et al*., 2017), complex processes for authentication and quality monitoring along their value chains are needed (Bent, 2008; Gertsch, 2009; Heinrich, 2015; Booker *et al*., 2015). Furthermore, a challenge in herbal pharmacovigilance is the development of novel approaches to monitor the safety of commercialised products (Barnes, 2003; de Boer *et al*., 2015). In this review we discuss the benefits and limitations of the biological identification and authentication methods, DNA barcoding and DNA metabarcoding, and show their potential in improving the quality control procedures of drug substances and resulting herbal products. The production chains of regulated or unregulated herbal medicinal products result in different requirements at the various production stages. Consequently, different analytical (chemical and biological) methods will have different roles in quality control and authentication (Figure 1).

## Regulatory status of herbal products

Herbal product regulation varies between countries. In the European Union/European Economic Area (EU/EEA), herbal products fall into two main categories, herbal medicines and herbal food supplements (botanicals), depending on their primary intended use. The EU directive 2004/24/EC, commonly referred to as the Traditional Herbal Medicinal Products Directive (THMPD) establishes a simplified procedure allowing the registration of herbal medicines as medicinal products (European Commission, 2004). The regulation applies to herbal products with a longstanding tradition of use, that have been on the market for at least 30 years, including 15 years in an EU member state. No special assays or trials are required to prove the safety of herbal medicines, but proof that the product is not harmful is compulsory (European Commission, 2004; Jütte *et al*., 2017). Since 2004, the EU Directive 2004/24/EC has been incorporated into national legal systems to increase the safety of traditional herbal medicines and to facilitate their free movement by harmonising previous national regulatory frameworks (Vlietinck *et al*., 2009; Jütte *et al*., 2017). The Herbal Medicinal Products Committee (HMPC) is the European Medicines Agency's (EMA's) committee responsible for establishing monographs on the therapeutic uses and the safety of medicinal products, with the pharmacovigilance of the marketed herbal medicines being a regulatory requirement falling under the full responsibility of manufacturers and suppliers.

The EU directive 2002/46/EC defines food supplements as concentrated sources of nutrients or other substances with nutritional or physiological effects, whose purpose is to supplement the normal diet (European Commission, 2002). Herbal food supplements are governed by the legislation of each EU's member state, and their quality and safety need to fulfill the requirements of food legislation, which are considerably less stringent than those for medicines. The safety and conformity with the food law requirements is under the full responsibility of manufactures and suppliers.

As a result of these distinct legislative frameworks, the same herbal product may coexist under different categories. For instance, Ginkgo biloba L. is regulated as a medical product in Germany, as a traditional herbal medical product or a food supplement in the UK, and in other European countries, Russia and the United States as a food supplement (Heinrich, 2015). Similar regulatory differences apply also to Hypericum perforatum, for example, L. (St John's wort), *Echinacea* sp. (coneflowers), Actaea racemosa L. (black cohosh), Eleutherococcus senticosus (Rupr. & Maxim.) Maxim. (Siberian ginseng).

## Quality issues and analytical challenges

One of the core interests of modern pharmacognosy refers to the identification and authentication of drug substances and to the quality of the resulting herbal medicines (Heinrich *et al*., 2017). However, the complex natural formulations, as well as the lack of standardised operating procedures and analytical methods, complicate the quality control of the herbal products. The identification and authentication of raw plant material and final herbal products relies on sensory and phytochemical screening techniques to detect species‐specific characters and compounds respectively (EMA, 2006; World Health Organisation (WHO), 2011; EDQM, 2014). The European Pharmacopoeia (Ph. Eur.) is the cornerstone of the quality control of raw materials and herbal products, that determines analytical procedures to be applied for qualitative and quantitative assays (EDQM, 2014; Agapouda *et al*., 2017). Ph. Eur. contains monographs with recommendations of analytical procedures for more than 200 primary materials (botanical drugs) and in some cases the resulting extracts (EDQM, 2014). However, herbal products are usually complex matrices and formulations, resulting from manifold processing steps, that pose unique challenges to the identification and authentication of raw material using organoleptic, morphological or microscopic features and standard chemical analytical assessments to determine species‐specific target compounds (Khan and Smillie, 2012). The various factors that may influence the quality of the raw material and resulting herbal products need to be carefully taken into consideration when determining the analytical method of choice for the authentication and quality control. Factors, as for instance the secondary metabolites in plants, are prone to variability under natural conditions from factors including age, seasonality, latitude, altitude, soil conditions, and herbivory, with direct influence on the concentration of the lead or marker compounds measured in different batches of raw plant material. This complex production and processing will consequently be reflected in the quality of the final herbal product (Zhang *et al*., 2012). Also, the more complex herbal products contain numerous ingredients, often extracted and processed differently, thus hampering an accurate authentication and quality control (Zhang *et al*., 2012; Bilia, 2014). Difficulties in the quality assurance processes arise also from some Pharmacopoeias around the world allow more than one plant species as a source for a botanical drug, or often assign similar functions in treating certain diseases. For instance, 140 out of 551 botanical drugs included in the 2015 edition of the Chinese Pharmacopoeia have multiple sources (Zhao *et al*., 2006; Wenzig and Bauer, 2009). Safety issues in the herbal products industry arise from the deliberate use of adulterants and admixture with undeclared fillers, in order to reach a certain chemical level or expected effect (Ko, 1998; Song *et al*., 2000; Ernst, 2002; Yee *et al*., 2005; Esters *et al*., 2006; Miller and Stripp, 2007; Wenzig and Bauer, 2009; Shewiyo *et al*., 2012). Most likely, the fraudulent use of fillers and plant materials of inferior quality is driven by the increasing level of consumption of herbal products which exceed the supply capacity for some plant species. Accidental substitutions leading to an improper utilisation of a botanical drug also often occur in the herbal products industry. For instance, plant identification relying on morphological characters of the plant species, or on standard analytical instrumental methods, may lead to misidentification in case of phenotypic plasticity or morphologically cryptic taxa, which often occur in some plant groups (Bickford *et al*., 2007). Similarly, challenges are posed by inconsistencies among vernacular names, pharmaceutical names, scientific synonyms or incorrect use of scientific generic names of the raw material (Wu *et al*., 2007; Ouarghidi *et al*., 2012; Walker and Applequist, 2012; Bennett and Balick, 2014; Saslis‐Lagoudakis *et al*., 2015; de Boer *et al*., 2015). The risks from unreported ingredients used in the herbal products may range from simple misleading labelling to potentially serious adverse drug reactions (Ernst, 1998; Heubl, 2010; Gilbert, 2011) or poisoning due to toxic contaminants (Chan, 2003). In summary, along their entire value chain, from cultivation or harvesting of the medicinal plants to the final marketed herbal product, a plethora of factors may directly influence the quality (Zhang *et al*., 2012).

## Developments in analytical methods

Despite major advances in the development of new analytical approaches, there is still a significant gap in quality control strategies that are applied to herbal products. For industrial analysis, emphasis has been placed on using single quick and thus cost‐effective techniques (i.e. TLC, HPTLC or HPLC) for primary qualitative analysis, or alternatively using hyphenated methods (i.e. HPLC‐UV, HPLC‐DAD, HPLC‐MS, GC‐MC, or LC‐NMR) to enable also the quantification of the lead or marker compounds (Patel *et al*., 2010; Zhang *et al*., 2017). Combining phytochemical and metabolomics approaches has been suggested for quality control and authentication in herbal value chains, especially of starting materials (Booker *et al*., 2012, 2014, 2016a, 2016b). Developments in DNA sequencing have spurred the fields of DNA barcoding and DNA metabarcoding, two approaches of increasing relevance for authentication of herbal ingredients and products (de Boer *et al*., 2015; Ichim *et al*., 2016; Raclariu *et al*., 2017a, 2017b).

## DNA barcoding and metabarcoding

The use of DNA barcoding enables species‐level identifications using short standard DNA regions, known as DNA barcodes (Hebert *et al*., 2003). DNA barcoding is widely applied by the scientific community and industry for molecular identification to solve a broad range of questions in taxonomy, molecular phylogenetics, population genetics, and biogeography (Hebert and Gregory, 2005; Hajibabaei *et al*., 2007; Valentini *et al*., 2009), as well as in trade control to prevent illegal wildlife collection and trade of flora and fauna (Chen *et al*., 2008; Eurlings *et al*., 2013; Gathier *et al*., 2013; Ghorbani *et al*., 2015; Janjua *et al*., 2016) and food product authenticity monitoring (Wong and Hanner, 2008; Yancy *et al*., 2008; Hanner *et al*., 2011; Cline, 2012; Di Pinto *et al*., 2016). In recent years in the field of medicinal plants research on DNA barcoding remarkable progress has been made, as reviewed by Techen *et al*. (2014) and de Boer *et al*. (2015). Initially used as an identification tool, DNA barcoding is now applied in the industrial quality assurance context to authenticate a wide range of herbal products (de Boer *et al*., 2015; Parveen *et al*., 2016; Sgamma *et al*., 2017). Recently, the British Pharmacopoeia included the first globally general DNA‐based identification method using Ocimum tenuiflorum L. (Lamiaceae), with the focus on plant sampling, barcode regions, DNA extraction, purification and amplification, and the sequences reference database (Heinrich *et al*., 2017; Sgamma *et al*., 2017). Recent investigations applied DNA barcoding to identify and authenticate various marketed herbal products, reporting various degrees of discrepancy between the expected species and the actual identified species. For instance, significant substitution was found in 98% of products of the traditional Chinese medicine (TCM), Baitouweng, which are expected to contain *Pulsatilla chinensis* (Bge.) Regel (Shi *et al*., 2017), 26% of single ingredient products purchased from local markets in Iran (Ghorbani *et al*., 2017), 16% of ginkgo herbal dietary supplements sold as dried and powdered leaves, purchased on‐line and in retail stores from the New York area (Little, 2014), 7% of *Senna* and 50% of *Cassia* market products in India (Seethapathy *et al*., 2014), 6% of saw palmetto herbal dietary supplements sold as dry, cut, gelatine capsules and compression tablets, purchased on‐line and at retail stores in the New York area (Little and Jeanson, 2013), 25% of black cohosh dietary supplements purchased on‐line and at retail stores in the New York area (Baker *et al*., 2012), 50% of Korean ginseng natural health products capsules, sold as tablets, roots, carved roots, extracts, teas and dried and shredded products, purchased from various commercial sources, including pharmacies and markets in Toronto and New York (Wallace *et al*., 2012), 35% of herbal tea products purchased from 25 different locations in the New York area (Stoeckle *et al*., 2011); and in 59% of herbal products sold as capsules, powders and tablets, purchased from the Toronto area or on‐line from distributors in the United States (Newmaster *et al*., 2013).

In the wake of these studies and of other recent cases exposing discrepancies between labelling and constituents actually present in the products, serious concerns were raised about the authenticity and quality of herbal products. Sgamma *et al*. (2017) discusses the feasibility and the main aspects of using DNA barcoding in industrial quality assurance procedures. However, conventional DNA barcoding faces practical limitations restricting the method to the authentication of a single ingredient herbal preparations exclusively, and only for unprocessed plant material thus before the plant undergoes various extractions and processing steps that usually lead to loss, degradation or mixing of DNA.

The combination of high‐throughput sequencing (HTS) and DNA barcoding, known as DNA metabarcoding, enables simultaneous high‐throughput multi‐taxa identification by using the extracellular and/or total DNA extracted from complex samples containing DNA of different origins (Taberlet *et al*., 2012; Staats *et al*., 2016). DNA metabarcoding is applicable to identification of plant species diversity in a range of products and has been used to investigate the level of discrepancy between the expected and detected plant species based on the label claims of marketed herbal products (Cheng *et al*., 2014; Coghlan *et al*., 2015; Ivanova *et al*., 2016; Raclariu *et al*., 2017a, 2017b). For instance, Coghlan *et al*. (2012) found that 15 highly processed TCMs contained species and genera included on CITES appendices I and II. Ivanova *et al*. (2016) found that 15 tested herbal supplements contained non‐listed, non‐filler plant DNA. The quality of 27 tested herbal preparations was highly affected by the presence of contaminants (Cheng *et al*., 2014). Out of 78 Hypericum perforatum herbal products only 68% contained the target species and detected divergence between constituent species and those listed on the label in all products (Raclariu *et al*., 2017b). Only 15% of investigated *Veronica* herbal products contained the target species Veronica officinalis L., whereas the main known adulterant, Veronica chamaedrys L., was detected in 62% of the products (Raclariu *et al*., 2017a).

All these studies report varying degrees of authentication success. Therefore, obtaining a representative assessment of complex herbal mixtures is influenced by many factors, including the quality and type of raw material, as well as several elements of the analysis that can however be varied to optimise the results (Staats *et al*., 2016). Some limitations of DNA metabarcoding are similar to those found in DNA barcoding. For instance, such methods may provide positive authentication of plant ingredients based on the presence of any amplifiable DNA, and false negatives can be expected if the DNA has been degraded or lost during post‐harvest processing or manufacturing (de Boer *et al*., 2015).

In the context of the quality control of herbal products, DNA barcoding and metabarcoding do not provide any quantitative nor qualitative information of the active metabolites in the raw plant material or the resulting preparation, and this narrows its applicability only to identification and authentication procedures. However, the use to identify and discern taxa at any developmental or processed stage from which DNA can be extracted is an essential advantage of DNA barcoding and metabarcoding (Hebert *et al*., 2003; Hajibabaei *et al*., 2007). The greatest advantage of DNA metabarcoding is its ability to identify each single species within complex multi‐ingredient and processed mixtures simultaneously, where the application of DNA barcoding and conventional analytical methods is limited considerably. Importantly, DNA metabarcoding data is usable for qualitative evaluation only, to determine presence of taxa, and not for quantitative assessment of relative species abundance based on sequence read numbers, as many variables considerably impact the obtained sequence read results (Staats *et al*., 2016). Clearly, in the context of pharmacognosy and pharmacovigilance, a combination of analytical methods is unavoidable for comprehensive authentication and quality control of raw material and resulting products (Shetti *et al*., 2011; Mishra *et al*., 2016; Parveen *et al*., 2016; Heinrich *et al*., 2017; Pawar *et al*., 2017), and DNA‐based approaches offer important novel insights.

## Perspectives

Both chemical and biological methods require comparison of detected compounds against reference standards. For chemical methods these can be lead or marker compounds, adulterants or more advanced computational scans of chemical databases. For biological DNA‐based methods there are similar advanced computational scans linked to well‐curated nucleotide sequence repositories. Chemical methods can be used in all stages for quality control and provide insights into the presence or absence of compounds defining a product. Biological methods based on DNA barcoding can be used for authentication of raw biological materials from cultivation or wild‐harvest at source or before manufacturing, but not accurately once the material is mixed with other biological material. Biological methods based on DNA metabarcoding can be used for authentication of finished products, post‐marketing control and pharmacovigilance, and provide insight into the total species diversity in a product.

Each method has its benefits and limitations, and its specific strength when applied correctly in the herbal product value chain. Both DNA barcoding and metabarcoding have potential in the context of quality control of both well and poorly regulated supply systems. DNA barcoding can be used for authenticating products based on single herbal ingredients and DNA metabarcoding for assessment of species diversity in processed products, and both methods should be used in combination with appropriate hyphenated chemical methods for quality control. Standardisation of protocols for DNA barcoding and DNA sequence‐based identification are necessary before DNA‐based biological methods can be implemented as routine analytical approaches and approved by the competent authorities for use in regulated procedures.
